# Supplementary material for: Incorporation of Zinc Oxide Nanoparticles Biosynthesized from Epimedium brevicornum Maxim. into PCL Nanofibers to Enhance Osteogenic Differentiation of Periodontal Ligament Stem Cells
Source: Materials (Basel). 2025 May 15;18(10):2295. doi: 10.3390/ma18102295 (PMC12113110; doi:10.3390/ma18102295)
Supplement: Supplementary file 1 [file materials-18-02295-s001.zip › materials-3538006-supplementary.pdf]

A

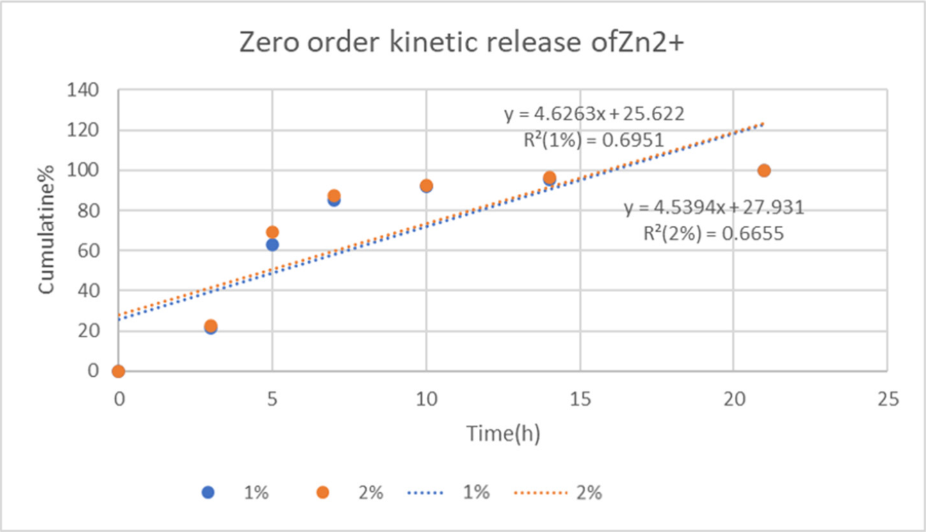
Zero order kinetic release ofZn2+
Cumulatine%
140
120
100
80
60
40
20
0
0
5
10
15
20
25
Time(h)
y = 4.6263x + 25.622
R²(1%) = 0.6951
y = 4.5394x + 27.931
R²(2%) = 0.6655
1%
2%
1%
2%

B

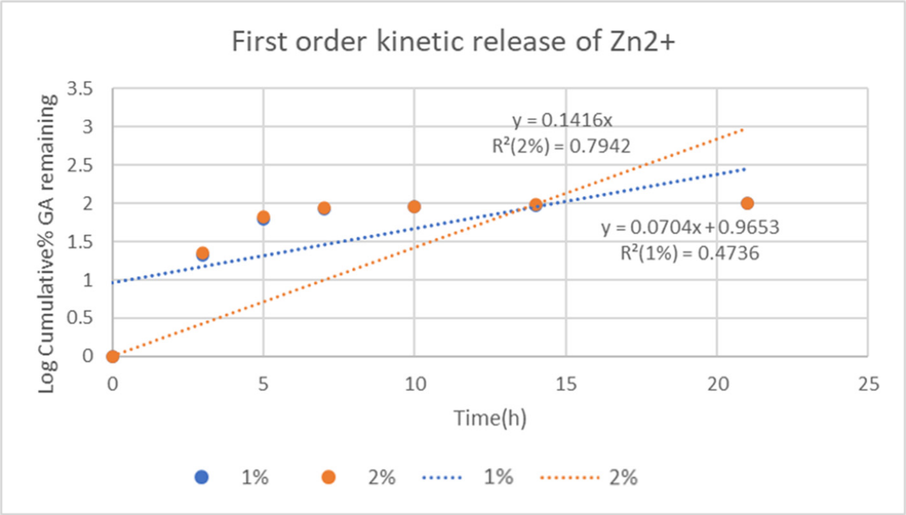
First order kinetic release of Zn2+
Log Cumulative% GA remaining
3.5
3
2.5
2
1.5
1
0.5
0
0
5
10
15
20
25
Time(h)
y = 0.1416x
R²(2%) = 0.7942
y = 0.0704x + 0.9653
R²(1%) = 0.4736
1%
2%
1%
2%

C

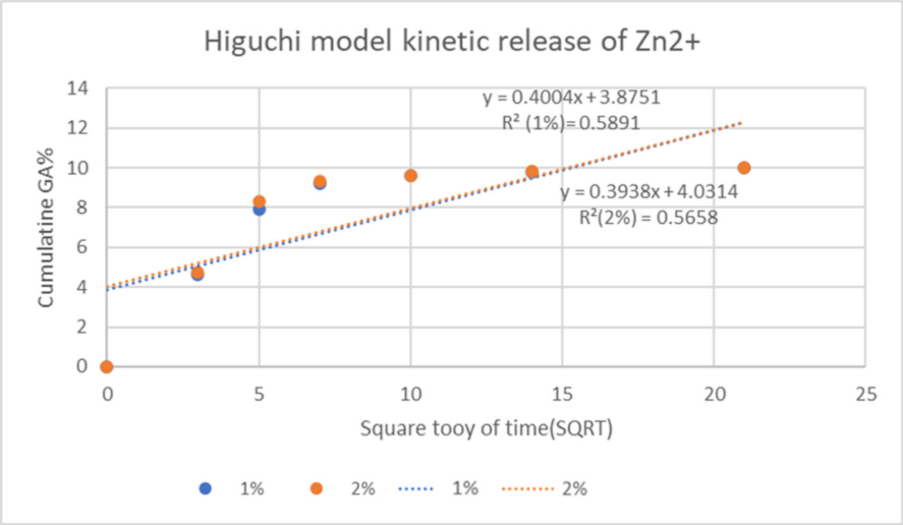
Higuchi model kinetic release of Zn2+
Cumulatine GA%
14
12
10
8
6
4
2
0
0
5
10
15
20
25
Square tooy of time(SQRT)
y = 0.4004x + 3.8751
R² (1%)= 0.5891
y = 0.3938x + 4.0314
R²(2%) = 0.5658
1%
2%
1%
2%

D

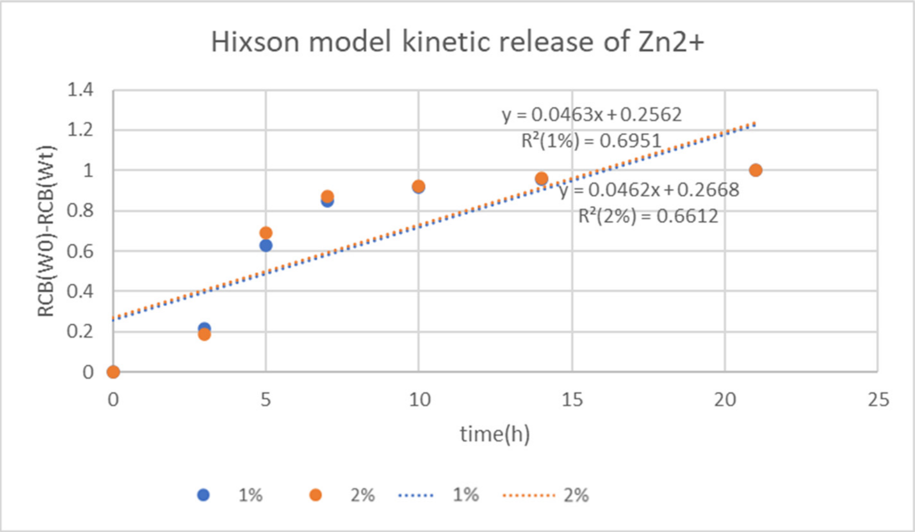

E

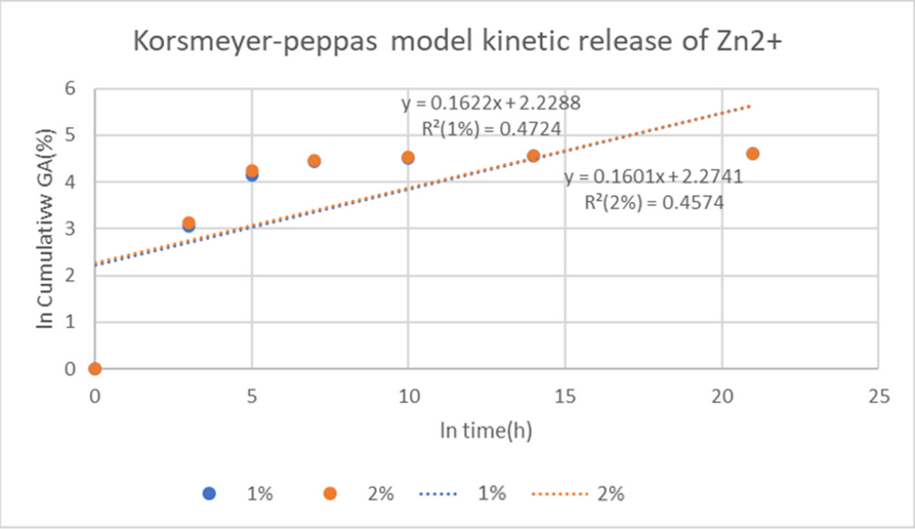

Figure S1. Fitting the data of zinc ion release from PCL nanofiber for kinetic models: Zero-order model (A), First-order model (B), Higuchi model (C), Hixson–Crowel model (D), and Korsmeyer–Peppas model (E).
